# The 18 kDa Translocator Protein (TSPO) Overexpression in Hippocampal Dentate Gyrus Elicits Anxiolytic-Like Effects in a Mouse Model of Post-traumatic Stress Disorder

**DOI:** 10.3389/fphar.2018.01364

**Published:** 2018-11-23

**Authors:** Xiao-Ying Zhang, Wang Wei, You-Zhi Zhang, Qiang Fu, Wei-Dong Mi, Li-Ming Zhang, Yun-Feng Li

**Affiliations:** ^1^State Key Laboratory of Toxicology and Medical Countermeasures, Beijing Institute of Pharmacology and Toxicology, Beijing, China; ^2^Anesthesia and Operation Center, Chinese PLA General Hospital, Beijing, China; ^3^Department of Anesthesiology, The General Hospital of the PLA Rocket Force, Beijing, China

**Keywords:** TSPO, post-traumatic stress disorder, neurosteroid, neurogenesis, hippocampus

## Abstract

The translocator protein (18 kDa) (TSPO) recently attracted increasing attention in the pathogenesis of post-traumatic stress disorder (PTSD). This study is testing the hypothesis that the overexpression of TSPO in hippocampus dentate gyrus (DG) could alleviate the anxiogenic-like response in the mice model of PTSD induced by foot-shock. In this study, hippocampal DG overexpression of TSPO significantly reversed the increase of the contextual freezing response, the decrease of the percentage of both entries into and time spent in the open arms in elevated plus maze test and the decrease of the account of crossings from the dark to light compartments in light–dark transition test induced by electric foot-shocks procedure. It was further showed that the behavioral effects of TSPO overexpression were blocked by PK11195, a selective TSPO antagonist. In addition, the expression of TSPO and level of allopregnanolone (Allo) decreased in the mouse model of PTSD, which was blocked by overexpression of TSPO in hippocampal dentate gyrus. The difference of neurogenesis among groups was consistent with the changes of TSPO and Allo, as evidenced by bromodeoxyuridine (BrdU)- positive cells in the hippocampal dentate gyrus. These results firstly suggested that TSPO in hippocampal dentate gyrus could exert a great effect on the occurrence and recovery of PTSD in this animal model, and the anti-PTSD-like effect of hippocampal TSPO over-expression could be at least partially mediated by up-regulation of Allo and subsequent stimulation of the adult hippocampal neurogenesis.

## Introduction

Post-traumatic stress disorder (PTSD) is a chronic and debilitating mental disorder that develops in survivors of traumatic events and PTSD can cause disturbing thoughts, helpless, and the attempt to avoid trauma-related cues (Bisson et al., 2015). However, the precise mechanisms of the intricate biological and psychological symptoms of PTSD remain unclear. Over the last two decades, the down-regulation of neurosteroid biosynthesis has been shown in several mental disorders including PTSD (Ceremuga et al., 2013; Locci and Pinna, 2017). Additionally, quite many clinical trials have shown that the decreased level of neuroactive steroids allopregnanolone (Allo) may play an important role in the pathology of PTSD (Rasmusson et al., 2006; Pinna, 2010; Pinna and Rasmusson, 2012). Pre-clinical studies have also found that corticolimbic Allo content remarkably reduced in patients of anxiety and aggression and the Allo decrease was positively related with the impaired behavioral performance (Pibiri et al., 2008; Pinna and Rasmusson, 2012). Previous results showed that the infusion of Allo into the dorsal hippocampus induced vigorous anxiolytic-like behavior in the elevated plus-maze test (Modol et al., 2011). These interesting studies gave reason to the hypothesis that downregulation of neurosteroids could contribute to the etiology of PTSD.

In the central nervous system, the translocator protein (18 kDa) (TSPO) are mainly expressed in glial cells. It mediates the translocation of cholesterol from the outer to the inner mitochondrial membrane, and thus regulates the synthesis of neurosteriods (Nothdurfter et al., 2012; Hatty and Banati, 2015). Studies demonstrated that long-term stress induced a decrease in the TSPO expression in the central nervous system in rodents (Milenkovic et al., 2015; Wang et al., 2015; Zhang et al., 2016). Interestingly, our group was the first to show that oral administration of certain TSPO ligands, including AC-5216 and YL-IPA08, enhanced synthesis of neurosteroids (such as Allo) in the brain and exerted anti-PTSD-like effect in some PTSD animal models with a favorable side effect (Qiu et al., 2013; Zhang et al., 2014a, 2016). Hence, TSPO protein might provide a promising target for novel anti-PTSD drug, but the specific mechanism remains to be determined.

The prefrontal cortex, the amygdala and the hippocampus are three brain regions in the limbic system which have been identified as the most clearly involved regions in PTSD (Wingenfeld and Wolf, 2014). Among them, the hippocampus plays an important role in remembrance of traumatic events and correlation of learned responses to contextual cues. Indeed, hippocampal reduction was reported to happen in patients with PTSD in many structural neuroimaging studies (Rodrigues et al., 2011). We therefore proposed that an up-regulation of TSPO expression in hippocampus, which could then enhance neurosteroidogenesis (such as Allo), may contribute to the behavioral adaptation to PTSD. To address our hypothesis, we used foot-shock procedure, an established mice model of PTSD to specifically examine the role of TSPO in PTSD (Bali and Jaggi, 2015). Furthermore, we examined a possible interference in PTSD-like behavior after application of a lentivirus-mediated overexpression of TSPO into the dentate gyrus (DG) of hippocampus. To explore the possible mechanisms in mediating the anti-PTSD effect of hippocampal TSPO overexpression, we then tested the changes of Allo and the hippocampal neurogenesis after behavioral tests.

## Materials and Methods

### Animals

Adult male ICR mice (18–22 g) were obtained from the Beijing SPF Animal Technology Company (Animal License No. SCXK 2016-0002; Beijing, China). All animals were housed in groups of 3 to 5 per plastic cage (320 mm × 220 mm × 160 mm) in an air-conditioned room of controlled temperature (23 ± 1°C) and a 12-h light/dark circle (lights on at 6:00 AM). Mice had access to food and water *ad libitum*. All procedures were conducted according to the National Institutes of Health Guide for the Care and Use of Laboratory Animals (8th edition). The experimental procedures were approved by the Institutional Committee on Animal Care and Use of Academy of Military Medical Sciences (No. IACUC.20094).

### Drugs and Treatments

Lentiviral vectors containing the non-targeting negative control (Lv-NC) or TSPO (Lv-TSPO) sequence were generated by Genechem Company (Genechem, Co., Ltd., Shanghai, China). Recombinant and packaging lentiviruses encoding the TSPO gene was constructed as our previous studies by the Genechem Company (Genechem, Co., Ltd., Shanghai, China) (Wang et al., 2016; Li et al., 2017). EGFP was added to all viral vectors to track lentivirus-mediated target gene as a marker expression. TSPO is expressed in different organs, including the adrenal cortex, luteal cells, the testis, ovarian granulosa cells, the placenta and glial cells in the brain. Specifically, we overexpressed TSPO in the bilateral hippocampus using a lentivirus to study the important role of TSPO in the hippocampal dentate gyrus and confirm the overexpression site by tracking the lentivirus-mediated target gene under an inverted fluorescence microscope.

**FIGURE 1:**
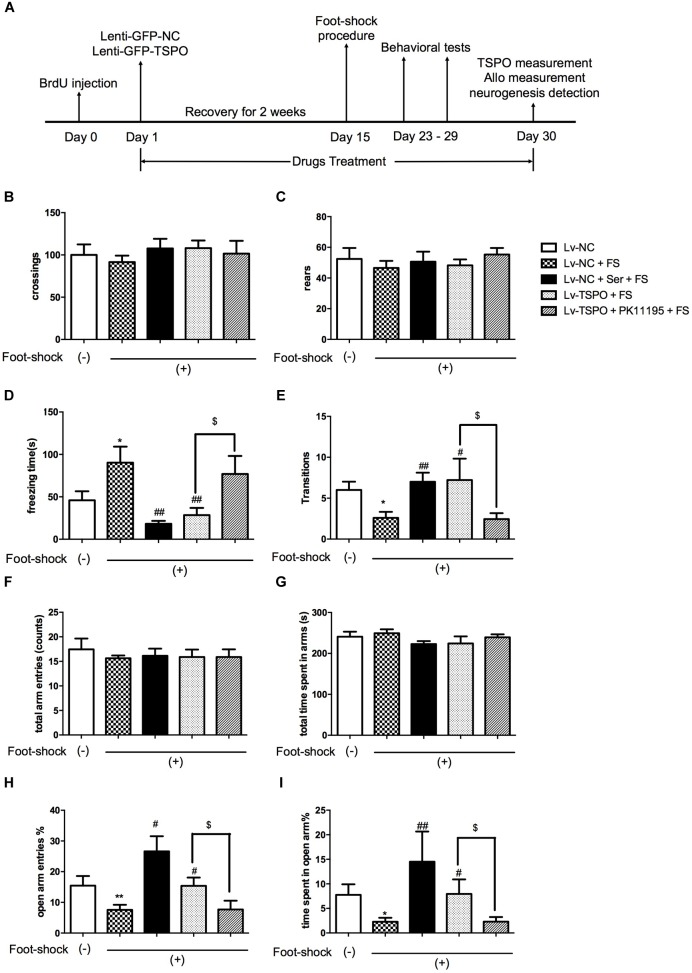
TSPO overexpression in the DG elicited anxiolytic-like effect in the mouse exposed to electric foot-shocks. **(A)** Design of the experiment. Results of spontaneous locomotor activity showed no difference among groups for the number of line crossings **(B)** or rears **(C)** in the open field test. **(D)** The contextual freezing time was increased in electric foot-shock model group (LV-NC + FS). The freezing behavior was significantly reduced in both Lv-NC + Ser + FS and Lv-TSPO + FS groups on day 23. **(E)** Exposure to foot-shock resulted in less transitions in the Light–dark transition test, which was attenuated by Ser or Lv-TSPO on day 29. **(F–I)** No differences were observed between groups for total arm entries **(F)** or total time spent in all arms **(G)** in the EPM test on day 27. Compared to the control group, the Lv-NC + Foot Shock group showed decreased open arm entries percentage **(H)** and time percentage in open arms **(I)**. And the effects were reversed by either Ser administration or Lv-TSPO injection. All the effects of Lv-TSPO in **(D,E,H,I)** was blocked by PK11195. Data were presented as the means ± SEM (*n* = 8–11). ^∗^*P* < 0.05 compared with the Lv-NC+foot-shock (–) group; ^#^*P* < 0.05, ^##^*P* < 0.01 compared with the Lv-NC+FS group; ^$^*P* < 0.05 compared with the Lv-TSPO+FS group.

Bromodeoxyuridine (BrdU), Sertraline (Ser, a serotonin reuptake inhibitor) and PK11195 (a TSPO antagonist) were purchased from Sigma-Aldrich (St. Louis, MO, United States). Ser (15 mg/kg, Sigma, St. Louis, MO, United States) was administered by intragastric gavage (*i.g.*) and PK11195 (3 mg/kg) was administrated intraperitoneally (*i.p.*, suspended in saline containing 2% DMSO and 0.8% Tween 80 for injection). Both drugs were given daily from day 1 to day 30 as Figure 1A shows. Behavioral tests were performed 1 h after the Ser or PK11195 administration.

### Experiment Design

Sixty mice were randomly assigned to five groups: Lv-negative control (NC), Lv-NC + foot-shock (FS), Lv-NC + Ser + FS, Lv-TSPO + FS and Lv-TSPO + PK11195 + FS (*n* = 12 for each). A schematic overview of the experiment is depicted in Figure 1A. First, BrdU (100 mg/kg, i.p.) was administered for 3 times at a 3 h interval 24 h before lentiviral vector administration. Then animals were subjected to microinjection of lentiviral vectors containing the non-targeting negative control (Lv-NC) or TSPO (Lv-TSPO) into the DG of hippocampus. Following a recovery period of 2 weeks, we conducted the electric foot-shock procedures and assessed the behavioral effects of over-expression of TSPO on anxiety-like behaviors induced by the inescapable electric foot shock, an established mouse model of PTSD.

To observe and confirm the microinjection sites, three vector-treated mice in each group were randomly chosen and perfused transcardially following the behavioral experiments. The brains were removed, post-fixed and dehydrated. Serial coronal brain sections (30 μm thick) were cut. The microinjection sites and infected zones were defined by direct visualization with a fluorescence microscope (Olympus AX70 Provis, Center Valley, PA, United States) for the benefit of the green fluorescent protein (GFP) tag as described previously (Li et al., 2009). To detect the TSPO protein expression and allopregnanolone (Allo) level after hippocampus injection of Lv-NC or Lv-TSPO, hippocampal tissues (3 mm in diameter around the injection site on both sides) were removed and Western blot analysis (*n* = 3) and enzyme-linked immunosorbent assay (ELISA) (*n* = 3) were performed respectively as described previously. The neurogenesis in hippocampus DG was evaluated by the immunohistochemistry of BrdU/NeuN-positive cells in DG (*n* = 3).

### Mouse Surgery and Lentiviral Microinjections

After 2-week acclimatization period and the following BrdU administration, mice received lentiviral microinjection under anesthesia with chloral hydrate (400 mg/kg, *i.p.*). The craniotomy was created aimed for bilateral DG according to the coordinates of the mouse brain atlas (Franklin and Paxinos, 2008): (AP, -1.7 mm; ML, ±1.8 mm; DV, -2.0 mm). The bilateral DG were both injected by a 10 μl microsyringe on the stereotaxic apparatus. The 30-gauge-needle was lowered into the dorsal DG. Lentiviral vectors containing NC or TSPO (2 × 10^8^ TU/μl, 1 μl/side) were infused at a rate of 0.2 μl/min by a microsyringe injector and Micro4 controller (World Precision, Ins., Sarasota, FL, United States). The amount of lentivirus and the duration of infusion were determined by the repeated preliminary experiments and previous studies. The needle stayed in place after injection for 5 min to guarantee proper diffusion of the vectors. From the day (Day 1) of lentiviral microinjection, Ser (15 mg/kg, *i.g.*) or PK-11195 (3 mg/kg, *i.p*.) was administered once per day. The dosages were selected according to our previous studies (Miao et al., 2014; Zhang et al., 2014b; Qiu et al., 2015).

### Behavioral Experiments

#### Electric Foot-Shock Procedures

Two weeks after lentiviral vectors injection, the electric foot-shock procedure was conducted as per Zhang et al. (2012) and Qiu et al. (2013). Electric foot-shocks were delivered through a stainless-steel grid floor (9 mm interval) by an isolated shock generator (Med Associates, Inc., United States) in a Plexiglas chamber (20 cm × 10 cm × 10 cm). Each mouse received 15 intermittent inescapable foot-shocks (intensity: 0.8 mA, interval: 10 s, and duration: 10 s) for 5 min following a 5-min adaptation period. Mice in the control group were placed in the same chambers without electric foot-shocks for total 10 min to adapt to and remember the same circumstance without trauma. Ethyl alcohol was used to wipe the chamber to avoid the effect of feces and smell between mice.

#### Contextual Freezing Measurement

The rodents of electric foot-shock model will freeze intermittently when re-exposed to the shock context and the freezing behavior is associated with the fear memory induced by the trauma-related cues, as the symptoms of PTSD patients. Thus, contextual freezing measurement was reported as one of the effective methods to evaluate PTSD (Maier, 1990; Siegmund and Wotjak, 2007; Zhang et al., 2012). All mice were re-exposed to the same chamber, which is the reminder situation of foot-shocks for 5 min 8 days after electric foot-shock procedures (Day 23; Figure 1A). The total cumulative freezing time was recorded and analyzed by computer (Med Associates, Inc., Video Freeze SOF-843, United States).

#### Open Field Test

To evaluate whether the reversion of PTSD-like behavior by over-expression of TSPO depends on locomotor activity change in mice, the number of line crossings and rears were assessed 10 days after electric foot-shock procedures (Day 25; Figure 1A) as in our previous study (Qiu et al., 2013; Zhang et al., 2016). Mice were placed in the center of a transparent box (36 cm × 29 cm × 23 cm) of which the base were divided into nice equal section. And then an observer who was blind to the study design counted and recorded the number of crossings (all four paws placed into a new square) and rears (both front paws raised from the floor) for 5 min. Ethyl alcohol was used to wipe the interior wall and floor to avoid the effect of feces and smell between mice.

#### Light–Dark Transition Test

Twelve days after electric foot-shock procedures (Day 27; Figure 1A), each mouse started in the dark side of the light–dark chamber and was free to cross through an opening between the light compartment and the dark one (30 cm × 23 cm for each side). The illumination of the light compartment was 720 lux. This test pits mice’ innate aversion to bright areas against their natural drive to explore in response to mild stressors such as a novel environment. The transitions were only defined by the number of crossings from the dark to the light compartments. An observer who was blind to the study design counted and recorded the transitions for 5 min.

#### Elevated Plus Maze Test

Fourteen days after electric foot-shock procedures (Day 29; Figure 1A), the elevated plus maze test was performed. It is a widely-applied method to assess the anxiogenic-like behavior of PTSD model rodents (Li et al., 2009; Zhang et al., 2012). The four branching arms (30 cm × 5 cm) composed the cross-shaped maze. The maze was set at 40 cm above the ground. The opposite two open arms without wall and two closed arms with dark walls (10 cm high) were connected by a central platform (5 cm × 5 cm). Each mouse was placed on the central platform and recorded by video for the time spent on the open/closed arms and number of entries into any arm. The entrance was defined by all four paws on the arm base. We calculated the ratio of time spent in the open arms to the total time spent in all arms and the ratio of the number of entries into open arms to that into any arm. The maze was cleaned with 5% ethanol between tests to avoid the effect of feces and smell between mice.

### Western Blot Analysis

The western blot analysis was conducted as described previously (Wang et al., 2016). Briefly, hippocampal tissues (3 mm in diameter around both injection sites) were removed and then extracted by RIPA lysis buffer (Applygen, China). Fifty microgram of protein were separated by SDS-PAGE, measured and analyzed by western blot with primary antibody rabbit anti-TSPO (1:1000; Abcam, Cambridge, MA, United States) and β-actin (1:3000; Santa Cruz, CA, United States). The expression of protein was measured by Gel-Pro Analyzer software, Version 3.1 (Media Cybernetics, Rockville, MD, United States) and the TSPO expression was normalized to β-actin. Every experiment was independently repeated no less than four times.

### Enzyme-Linked Immunosorbent Assay (ELISA)

The removed hippocampal tissues (3 mm in diameter around both injection sites) were extracted on ice with the lysis buffer containing 137 mM NaCl, 0.5 mM sodium vanadate, 10 μg/mL aprotinin, 1 μg/mL leupeptin1% NP40, 10% glycerol, 1 mM PMSF, 20 mM Tris-HCl (pH 8.0). Allo concentrations were quantified using ELISA kits according to the manufacturer’s protocol (Arbor Assays, United States), and the density values were detected by the spectrophotometer at a wavelength of 450 nm and a reference wavelength of 650 nm.

### Immunohistochemistry

Immunohistochemistry was conducted as Li et al. (2009). Mice were anesthetized deeply with chloral hydrate (500 mg/kg, *i.p.*), transcardially perfused with ice-cold 0.9% NaCl and then 4% buffered formalin. Brains of mice that received intraperitoneal injections of BrdU were carefully and quickly removed and fixed in 4% paraformaldehyde at 22°C for 48 h for histochemistry of BrdU. Coronal 12-μm sections were cut and incubated free-floating for 24 h at 4°C in PBS containing both rat anti-BrdU antibody (1:200; Abcam, Cambridge, MA, United States) and mouse anti-NeuN antibody (1:1000; Chemicon, Temecula, CA, United States). After rinsing with PBS for three times, the sections were then incubated with Red-X-conjugated goat anti-rat IgG and FITC- conjugated goat anti-mouse IgG (1:200 for both; Jackson, MS, United States) to react to the corresponding primary antibody in PBS for 2 h at 22°C before mounting. The sections were photographed and analyzed by confocal microscope (Zeiss LSM510, Thornwood, NY, United States). The BrdU-positive cells were counted as described previously (Li et al., 2009). Briefly, the BrdU immunohistochemistry was performed for every sixth section throughout the entire hippocampus. All BrdU-positive cells in the hippocampus DG were counted by a blind observer and multiplied by 6, recorded as the total number of labeled cells in the DG.

### Statistical Analysis

Data were analyzed using GraphPad Prism 6 software (Graphpad Prism Institute, Inc., La Jolla, CA, United States). Results are expressed as means ± SEM. Outliers were removed according to the interquartile range (IQR) test (Zijlmans et al., 2018). Outliers here were defined as observations that fall below data set median - 1.5 IQR or above data set median + 1.5 IQR. Data were analyzed by Mann–Whitney *U*-test for multiple comparisons, followed by the Holm–Sidak test as *post hoc* analyses to adjust. Values of *P* < 0.05 were considered statistically significant.

## Results

### TSPO Overexpression in the DG Elicited Anxiolytic-Like Effect in the Mice Exposed to Electric Foot-Shocks

There was no significant difference in the line crossings and rears between groups in the open field test. These results indicated that none of Lenti, Ser (15 mg/kg) or PK11195 (3 mg/kg) significantly did harm to locomotor activity in this animal model (Figures 1B,C).

A significant increase in the contextual freezing time was observed in Lv-NC + Foot Shock group compared to the non-shocked Lv-NC group, indicating that the anxiogenic-like mouse model of PTSD was successfully established. The freezing behavior was alleviated in the Lv-NC + Ser + FS group as the positive control compared with Lv-NC + FS group. After Holm–Sidak correction was used to calibrate the error from multiple tests, the significant difference remained, demonstrating that the validity of this model (*P* = 0.0272 for Lv-NC+FS vs. Lv-NC; *P* = 0.0019 for Lv-NC+Ser+FS vs. Lv-NC+FS; Figure 1D). The contextual freezing response was also decreased in mice that received an intra-hippocampal injection Lv-TSPO compared with foot-shock vehicle group (*P* = 0.0038 for Lv-TSPO+FS vs. Lv-NC+FS; Figure 1D). These results demonstrated that TSPO overexpression in DG of hippocampus attenuated the contextual freezing behavior in post-shocked mice.

In the light–dark transition test, the number of crossings from dark to light compartment decreased in the foot-shock-exposed Lv-NC + FS mice compared to the non-shocked Lv-NC mice (*P* = 0.0134 for Lv-NC+FS vs. Lv-NC; Figure 1E). It was also shown that either repeated administrations of Ser or Lv-TSPO injection increased the number of transitions (*P* = 0.0018 for Lv-NC+Ser+FS vs. Lv-NC+FS; *P* = 0.0461 for Lv-TSPO+FS vs. Lv-NC+FS; Figure 1E), suggesting that TSPO overexpression in DG of hippocampus significantly ameliorated PTSD-associated anxiogenic-like behaviors.

As shown in Figures 1F,G, no significant differences were observed between groups for total arm entries or total time spent in all arms in the EPM test. Compared to the control group, both open arm entries percentage and time percentage in open arms decreased in the Lv-NC + FS group (*P* = 0.0249 for the number of entries into open arms; *P* = 0.0189 for percentage of time spent in open arms for Lv-NC+FS vs. Lv-NC). Compared with Lv-NC + FS group, Lv-TSPO treatment significantly increased the percentage of entries into open arms and the percentage of time spent in open arms (*P* = 0.0156 for the number of entries into open arms; and *P* = 0.0496 for percentage of time spent in open arms for Lv-TSPO+FS vs. Lv-NC+FS), as did repeated administration of Ser (*P* = 0.0013 for the number of entries into open arms; *P* = 0.0044 for percentage of time spent in open arms for Lv-NC+Ser+FS vs. Lv-NC+FS).

It was further showed that these above anti-PTSD behavioral effects of TSPO overexpression was reversed by PK11195 administration (*P* = 0.0412 for freezing time in contextual freezing test, Figure 1D; *P* = 0.0486 for dark-to-light transition in light–dark transition test, Figure 1E; *P* = 0.0355 for the percentage of entries into open arms, *P* = 0.0432 for percentage of time spent in open arms in EPM test, Figures 1H,I). These results indicated that TSPO overexpression in DG of hippocampus attenuated the anxiogenic-like behavior induced by electric foot-shock procedures in mouse model of PTSD, but these effects could be blocked by the TSPO ligand PK11195, suggesting that these effects might be at least partially attributed to TSPO activation.

### Targeted Overexpression of TSPO in the Hippocampus

**FIGURE 2:**
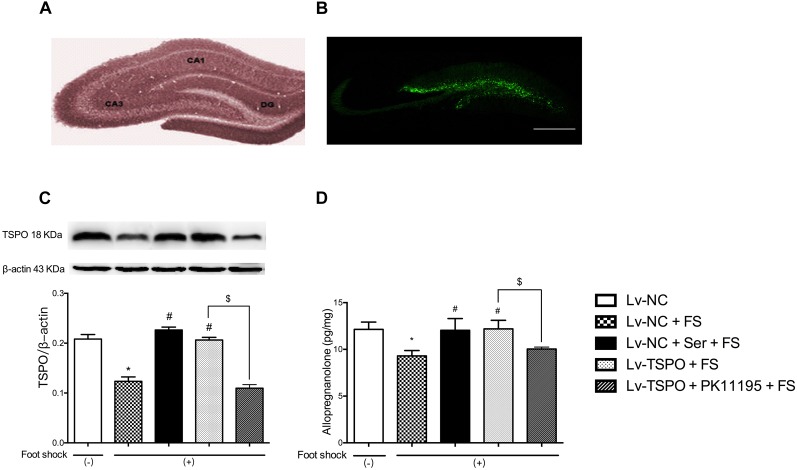
Effects of lenti-GFP-TSPO on expression of TSPO and level of Allo in the DG of mice. **(A,B)** Fluorescence microscopy captured the microinjection sites shown as expression of GFP in the DG; Scale bars = 500 mm; *n* = 3. **(C)** Representative immunoblots of TSPO and the histogram represents semi-quantitative results of western blot analysis. The protein expression of TSPO were normalized by β-actin; *n* = 3; **(D)** The level of Allo of hippocampal tissues; *n* = 3. Data were presented as the means ± SEM. ^∗^*P* < 0.05 compared with the Lv-NC+foot-shock (–) group; ^#^*P* < 0.05 compared with the Lv-NC+FS group; ^$^*P* < 0.05 compared with the Lv-TSPO+FS group.

To confirm the overexpression of TSPO *in vivo*, the lentivirus-mediated overexpression was traced by the expression of GFP using fluorescence microscopy (Figures 2A,B). The results showed that fluorescence obtained with this lentiviral vector was localized to the subgranular layer and hilus of the DG, as indicated by GFP-positive cells (green), while no expression was detected outside the hippocampus. Combined with the immunoblots of TSPO in punched hippocampus tissue, the results showed that foot-shock procedure significantly reduced the expression of TSPO (*P* = 0.0143), and chronic administration of Ser or Lv-TSPO injection clearly increased the TSPO expression (*P* = 0.0286 for Lv-NC+Ser+FS vs. Lv-NC+FS; *P* = 0.0143 for Lv-TSPO+FS vs. Lv-NC+FS) at the same time of exerting the anti-PTSD-like effect. The overexpression of TSPO by administration of LV-TSPO was blocked by TSPO antagonist PK11195 (*P* = 0.0143; Figure 2C).

### Effects of Hippocampal TSPO Overexpression on the Level Allo After Electric Foot-Shock

The endogenous Allo level in the hippocampus tissues (3 mm in diameter around both injection sites) of post-shock mice were measured at the end of the behavioral tests to further confirm the role of Allo in the anti-PTSD-like behavior effect of Lv-TSPO. As shown in Figure 2D, the foot-shock procedure significantly reduced the Allo level in the hippocampus compared to shock-free control mice (*P* = 0.0140), which was clearly reversed by daily administration of Ser or Lv-TSPO injection (*P* = 0.0475 for Lv-NC+Ser+FS vs. Lv-NC+FS; *P* = 0.0375 for Lv-TSPO+FS vs. Lv-NC+FS). And the increase of Allo by administration of LV-TSPO was blocked by TSPO antagonist PK11195 (*P* = 0.0312; Figure 2D).

### Effect of Hippocampal TSPO Overexpression on the Number of BrdU-Positive Cells in the Hippocampus in Mice After Electric Foot-Shocks

**FIGURE 3:**
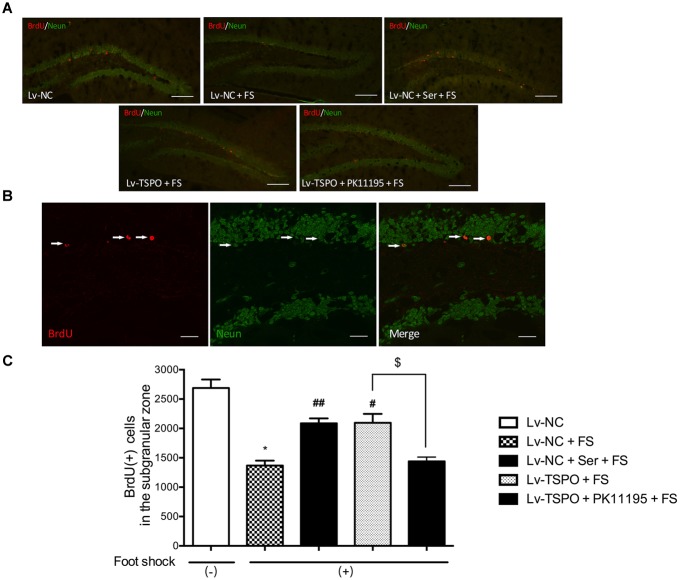
Effect of TSPO overexpression in the DG on the number of BrdU-positive cells in the hippocampus in mice after electric foot-shocks. **(A)** A confocal laser-scanning microscope micrographs of BrdU-labeled cells (red) in the dentate gyrus of hippocampus. Scale bar = 200 μm. **(B)** NeuN (green) and BrdU (red) co-labeled cells in the dentate gyrus were photographed and quantified under confocal microscope. Scale bar = 50 μm. **(C)** Statistical analysis showed a significant decrease of the number of BrdU-positive cells in mice of foot-shock exposure (LV-NC + FS) compared with non-foot-shocked mice (Lv-NC), whereas it was prevented in the group of Lenti-GFP-TSPO injection (Lv-TSPO +FS). The improvement of Lv-TSPO was reversed by PK11195 (Lv-TSPO + PK11195 +FS). Data were presented as the means ± SEM (*n* = 3). ^∗^*P* < 0.05 compared with the Lv-N+foot-shock (–) group; ^#^*P* < 0.05 compared with the Lv-NC+FS group; ^$^*P* < 0.05 compared with the Lv-TSPO+FS group. ^##^*P* < 0.01 compared with the Lv-NC+FS group.

Given the view that neurogenesis could be reduced by PTSD, we then labeled BrdU-positive cells in the hippocampus DG to determine the effect of TSPO on neurogenesis. Mice were sacrificed 30 day after the beginning of BrdU labeling. Cells labeled with BrdU were counted per bilateral, entire hippocampal dentate gyri. BrdU-positive cells were predominantly localized in the subgranular layer (Figure 3A) and co-localized with NeuN- cells (Figure 3B). Statistical analysis revealed that the foot-shock procedure significantly decreased the number of BrdU (+) cells present in the DG when compared with foot-shocked (-) mice (*P* = 0.0269 for Lv-NC+FS vs. Lv-NC). And this effect could be reversed by the chronic administration of Ser or Lv-TSPO injection which exhibited significantly more BrdU (+) cells than Lv-NC treated animals (*P* = 0.0085 for Lv-NC+Ser+FS vs. Lv-NC+FS; *P* = 0.0334 for Lv-TSPO+FS vs. Lv-NC+FS; Figure 3C). These data indicated that the overexpression of TSPO alleviated the impaired hippocampal neurogenesis induced by foot-shocked procedure.

## Discussion

In this study, we showed that Lv-TSPO mediated overexpression of TSPO in the DG attenuated PTSD-like behaviors without significantly affecting locomotor activity. These results suggested that overexpression of TSPO in the DG may reverse the PTSD-like behaviors, which can be reversed by a TSPO antagonist PK11195. Furthermore, hippocampal TSPO overexpression increased the level of Allo and improved hippocampal neurogenesis.

In the present study, the lentivirus was selected for its stable and long-term gene overexpression as the vector (Schratt et al., 2006; Krassnig et al., 2015; Chen et al., 2016). It was reported that lentiviral vectors can receive a big fragment of exogenous target gene, express constantly in cells, and possess satisfactory safety (Escors and Breckpot, 2010; Sauer et al., 2014). Till now, no studies about the application of lentiviral vectors to PTSD therapy were reported worldwide. Our previous study suggested that the lentiviral vectors carrying TSPO gene were well-expressed in the DG of hippocampus (Wang et al., 2016), which fits the evidence of our present study. As the lentiviral vectors efficiently inspired hippocampal TSPO signaling, it was examined whether TSPO expression change can modify the PTSD-like responses of mice after foot-shock paradigms, a reliable animal model for PTSD. Interestingly, our studies showed that intra-hippocampal injection of the Lv-TSPO reversed the behavioral impairment including the anxiogenic-like effect in mice after foot-shock exposure, which is consistent with the effect of chronic administration of sertraline. Sertraline, as a selective serotonin reuptake inhibitor (SSRI), is a FDA-approved medication for PTSD. It has shown its certain anti-PTSD effect independent on the shock exposure in many rodent models (Miao et al., 2014; Zhang et al., 2015; Qiu et al., 2017; Zhang Z.S. et al., 2017). Our results suggested that the dose of intra-hippocampal injection of the Lv-TSPO was efficient to exert the anti-PTSD effect as sertraline. We also found that neither the foot-shock procedure nor the intra-hippocampal injection of the Lv-TSPO significantly impacted on the locomotor activity in mice, suggesting that the observed behavioral differences were independent on the basal locomotor activity changes.

In order to investigate the anti-PTSD-like effect of intra-hippocampal injection of the Lv-TSPO, it was tested whether blocking the TSPO pathway by PK11195 affected the behavioral effects in the foot-shock procedure. According to our previous studies, chronic administration of PK11195 alone would not change the foot-shock induced PTSD-like behavior or the level of Allo in serum or brain (Zhang et al., 2014b). However, in this study when PK11195 intervened the effect of intra-hippocampal injection of Lv-TSPO in foot-shock model, it was shown that the TSPO antagonist PK11195 administration reversed all the attenuated behavioral effects induced by DG Lv-TSPO. These results were in line with our previous studies which demonstrated that PK11195 completely blocked the anti-PTSD-like effects of TSPO ligand YL-IPA08 (Zhang et al., 2013). Overall, these findings indicated that the anti-PTSD effects of intra-hippocampal injection of the Lv-TSPO could be mediated by TSPO activation.

The expression level of TSPO protein were measured and further verified the overexpression of TSPO in DG in the Lv-TSPO+FS group. The decreased TSPO expression induced by foot shock in PTSD mice model and increased TSPO expression by sertraline agree with our previous studies (Qiu et al., 2013; Zhang et al., 2014b; Li et al., 2017; Zhang L.M. et al., 2017). Interestingly, we found that PK11195 blocked the effects of TPSO over-expression on TPSO protein levels in the hippocampus. We think it might be some compensatory mechanism, for that TSPO is technically not a classic receptor. On the other hand, PK11195 was reported to be able to induce changes in expression of immediate early genes and transcription factors in U118MG glioblastoma cells which were studied for TSPO functions for years. These changes also included gene products that are part of the canonical pathway serving to modulate general gene expression (Yasin et al., 2017). It might be other reason for the TSPO expression change and needs further investigation.

Allo is the most abundant neurosteroid in the central nervous system potently and selectively acting with GABA_A_ the receptors and modulating of GABA_A_ signaling action (Puia et al., 1990; Lambert et al., 1995, 2003). The decreased level of Allo in the central nervous system was reported to associate with the symptoms of PTSD (Vaiva et al., 2004; Rasmusson et al., 2006). Numerous studies have demonstrated that Allo plays a pivotal role in the mediation of contextual fear memory cued by the trauma-related events and the incapacity of Allo biosynthesis may be one of the molecular mechanisms underlying the etiology of PTSD (Uzunova et al., 1998; Rasmusson et al., 2006; Pibiri et al., 2008; Pinna and Rasmusson, 2012). In this current study, we detected the level of Allo in mice to substantiate this hypothesis that the normalization of brain Allo levels may underlie the anti-PTSD-like effects of intra-hippocampal injection of the Lv-TSPO. Our results showed a remarkable decreased Allo level in the hippocampus in post-foot-shock mice, which was reversed by sertraline in hippocampus, and the result is in line with the previous knowledge that anti-PTSD-like activities of sertraline were closely associated with elevated biosynthesis of Allo (Pinna and Rasmusson, 2012; Xu et al., 2018). Likewise, the intra-hippocampal injection of the Lv-TSPO reversed the decreased Allo level in the hippocampus in post-foot-shock mice, further suggesting that the anti-PTSD-like effects of Lv-TSPO could be mediated by the subsequent synthesis of Allo in hippocampus DG. It is consistent with the results of previous studies on TSPO ligands, such as YL-IPA08 and AC-5216, which have been shown to display efficacy toward the treatment of psychiatric disorders by increasing neurosteroid biosynthesis in the brain, inducing anxiolytic and antidepressant activities in some rodent models and improving behavioral deficits in a mouse model of PTSD (Qiu et al., 2013; Zhang et al., 2014a; Wang et al., 2016; Li et al., 2017; Zhang L.M. et al., 2017). It is important to note that other neurosteroids were not examined in our present study, but their roles cannot be excluded and their potential contribution need further studies to identify. Our present study also found that sertraline could reverse the lowered Allo levels in post-foot-shock mice and further suggested that the anti-PTSD-like effects of sertraline were partly mediated by the subsequent synthesis of Allo.

A vast literature demonstrated the important role of adult-born neurons in buffering stress responses and in mediating anti-PTSD-like effects. Functional studies that have described the impaired adult hippocampal neurogenesis in PTSD patients and different animal models and the hippocampal neurogenesis became one of the treatment targets of anti-PTSD interventions (Kheirbek et al., 2012; Peng et al., 2013; Besnard and Sahay, 2016). This study showed that similar as sertraline, intra-hippocampal injection of the Lv-TSPO in post-foot-shock mice induced a significant proliferation of progenitor cells as shown by BrdU immunohistochemistry. Interestingly, evidences reported that exogenous administration of Allo could prevent the occurrence of depression/anxiety-like behavior, as well as alleviate the damage of hippocampal neurogenesis (Evans et al., 2012) and promote cell survival (Brinton, 1994; Djebaili et al., 2005). Furthermore, recent studies have demonstrated the neuroprotective role of Allo against the hippocampal neurogenesis impairment in a transgenic mouse model of Alzheimer’s disease (Wang et al., 2010; Singh et al., 2012). In addition, it was reported that besides GABAergic mechanisms, Allo could also enhance neurogenesis, which contribute to the regulation of depression and anxiety (Wang et al., 2005). These observations suggested that impaired hippocampal neurogenesis may participate the pathology of PTSD, and thus the hippocampal neurogenesis also provides a promising target for anti-PTSD treatment (Kempermann and Kronenberg, 2003).

In summary, the over-expression of TSPO in hippocampal DG exerted anti-PTSD effect in mice submitted to the foot-shock, which may be related to the up-regulation of Allo synthesis and subsequent stimulation of the adult hippocampal neurogenesis. It should also be stated that in this present study, we did not measure other neurosteroids or the new-born neurons in hippocampus DG, so we could not attribute the anti-PTSD effect of TSPO overexpression completely to increased Allo level. Nevertheless, our study advances our knowledge of the PTSD theory and provide a promising implication for the treatment of this mental disorder.

## Author Contributions

X-YZ helped to conceive the study, carried out the study execution and data analysis, and contributed to the manuscript draft. WW and QF contributed to the analysis of immunohistochemistry. L-MZ participated in the research design, the construction of recombinant lentiviruses, and the draft of manuscript. Y-ZZ, W-DM, and Y-FL contributed to the research design, data analysis, and manuscript revision.

## Conflict of Interest Statement

The authors declare that the research was conducted in the absence of any commercial or financial relationships that could be construed as a potential conflict of interest.
